# A Case of Digital Cutaneous Melanocytic Tumor With CRTC1::TRIM11 Fusion

**DOI:** 10.7759/cureus.33179

**Published:** 2022-12-31

**Authors:** Chau M Bui, Manita Chaum, Bonnie Balzer

**Affiliations:** 1 Pathology and Laboratory Medicine, Cedars-Sinai Medical Center, Los Angeles, USA

**Keywords:** melanocytic differentiation, melanocytoma, trim11, crtc1, cutaneous melanocytic tumor

## Abstract

A cutaneous melanocytic tumor with *CRTC1::TRIM11* fusion (CMTCT) was recently described as a novel superficial tumor with melanocytic differentiation and harboring a unique in-frame translocation, *CRTC1::TRIM11*. This emerging entity can occur at any age and is known to be a low-grade malignant neoplasm with limited follow-up data. There are no available guidelines for the management and treatment of this tumor. This neoplasm has been found in the extremities, head and neck, and trunk. Here, we present the first case occurring on acral digital skin. This case contributes to the growing knowledge surrounding this newly described entity.

## Introduction

Cutaneous melanocytic tumor with *CRTC1::TRIM11 *fusion (CMTCT) was first reported in 2018 [1]. To date, 47 cases have been reported in the literature, including the largest series of 41 cases comprising 32 new cases and nine previously reported cases published in November 2022 [1-8]. This entity can be seen in adults of all ages, and there have also been four cases described in children [2,3]. This tumor most commonly occurs in the dermis and subcutis, although there have been recent reports of cases located in the mucosa as well [3]. It presents as a small, usually painless, relatively well-circumscribed cutaneous nodule. It is dermally based without epidermal or subcutaneous involvement. It is composed of intersecting, short fascicles of the spindle or epithelioid cells with prominent nucleoli, and indistinct cell borders with melanocytic marker expression. Similar to other cutaneous melanocytic tumors, this entity also has a specific molecular feature, i.e., CMTCT. CMTCT is believed to be a low-grade malignant neoplasm. Most patients undergoing complete excision had a good prognosis without recurrence or metastasis [1-7]. Three out of 47 patients had local recurrence and metastasis after the initial resection [3,4,8].

## Case presentation

**Figure 1:**
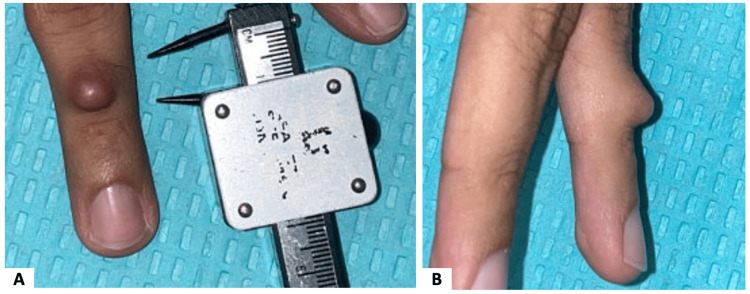
Clinical photos of CMTCT. CMTCT = *CRTC1::TRIM11* fusion.

Here, we present a case of a 27-year-old male with no significant history who presented with a 1.2 cm painless, slow-growing exophytic mass on his fourth finger. He noticed the mass 1.5 years prior. It was white, firm, and round, with normal-appearing overlying skin (Figure 1).

**Figure 2:**
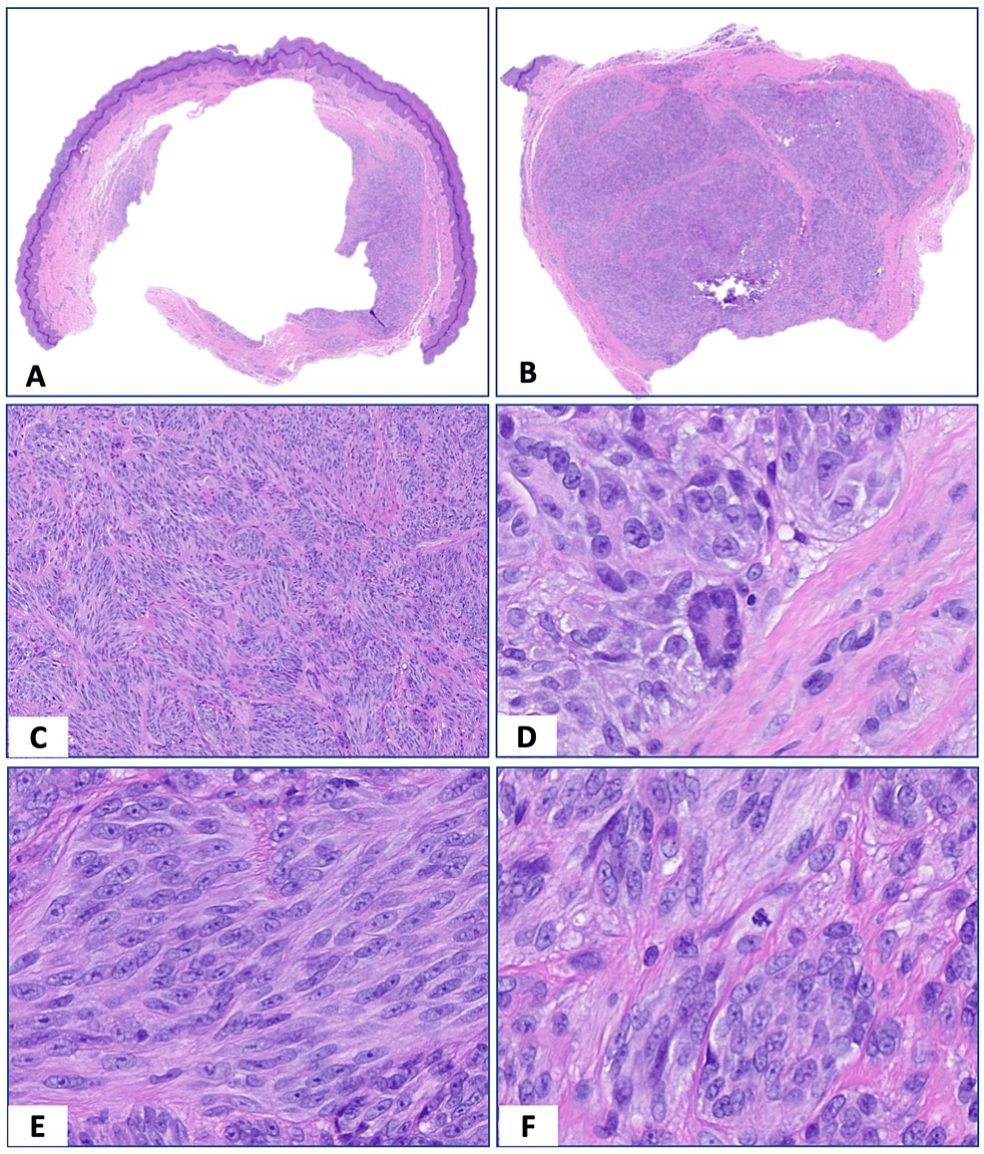
Histologic features of CMTCT. (A-B) Cellular tumor surrounded by a fibrous rim without dermal-epidermal junction involvement (H&E, 20x). (C) Cellular nests composed of intersecting short fascicles of monomorphic spindle cells separated by collagen (H&E, 40x). (D) Multinucleated giant cells (H&E, 400x). (E) Mid-sized uniform spindle cells with smooth to vacuolated chromatin, prominent nucleoli, slightly granular cytoplasm, and indistinct cell borders (H&E, 400x). (F) Abnormal mitosis (H&E, 400x). CMTCT = *CRTC1::TRIM11* fusion; H&E = hematoxylin and eosin.

**Figure 3:**
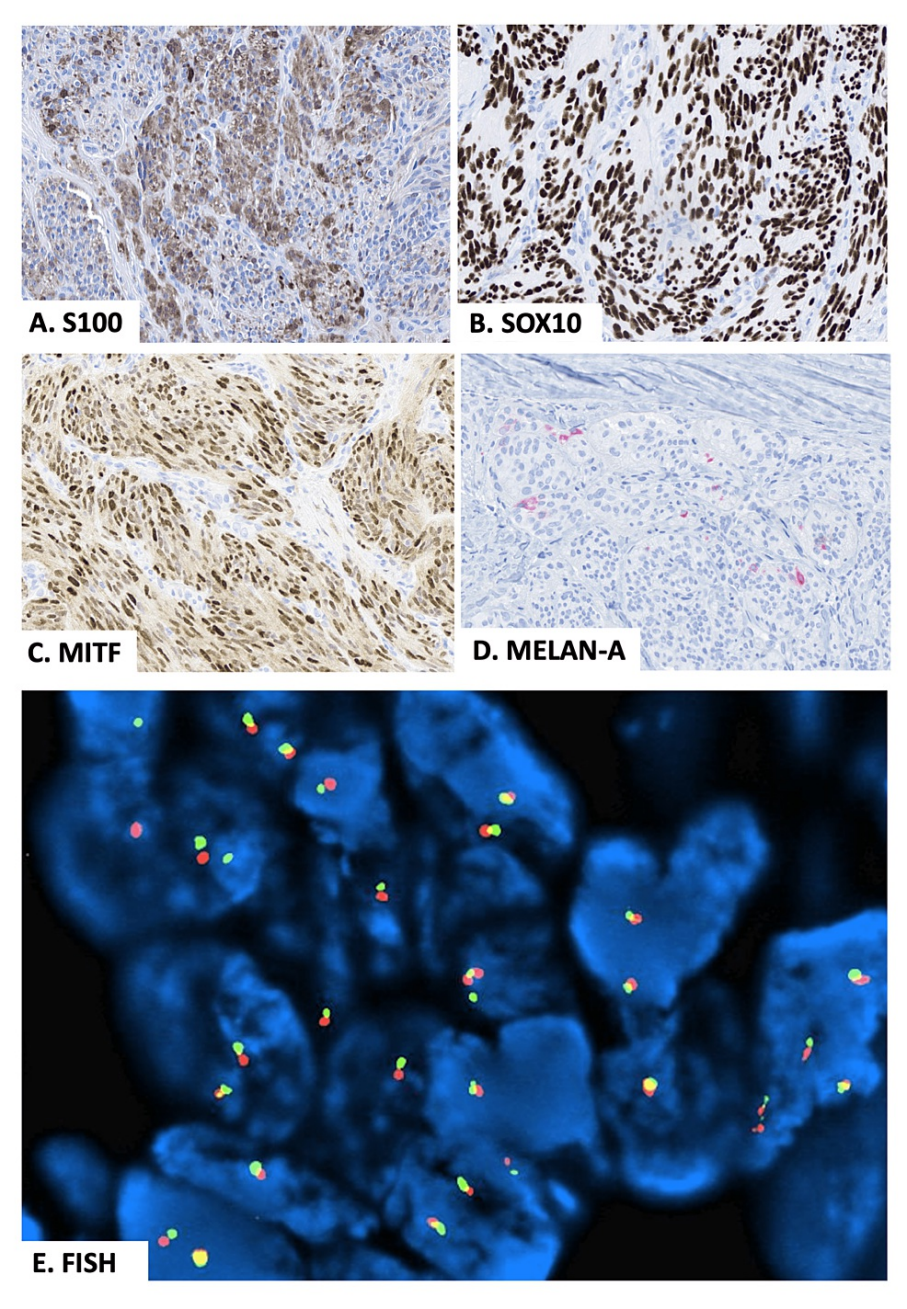
Immunohistochemical stains and fluorescence in situ hybridization (FISH) break apart of EWSR1 gene. (A) S100; (B) SOX10; (C) MITF; (D) Melan-1; (E) Negative FISH break apart of *EWSR1*.

An excisional biopsy was performed. Microscopic examination showed a dermal cellular tumor surrounded by a fibrous rim but without encapsulation or dermo-epidermal junction involvement (Figures 2A, 2B). The tumor was composed of discrete cellular nests and intersecting short fascicles of spindle cells with collagen in between (Figure 2C). At high power, there were mid-sized uniform spindle cells with smooth to vacuolated chromatin, prominent nucleoli, slightly granular cytoplasm, and indistinct cell borders (Figure 2E). Rare multinucleated giant cells and a few atypical mitoses were present (Figures 2D, 2F). Necrosis was absent. Tumor cells showed reactivity with melanocytic markers. S100 showed focal to patchy positivity (Figure 3A). SOX10 and MITF showed strong diffuse positivity, and Melan-A highlighted rare tumor cells (Figures 3B-3D). HMB45 was negative. Tumor cells were negative for factor-13A, CD34, smooth muscle actin (SMA), desmin, glucose transporter 1 (GLUT1), and epithelial membrane antigen (EMA), excluding soft tissue tumors with fibroblastic/fibrohistiocytic or myoid differentiation and nerve sheath neoplasms. Fluorescence in situ hybridization (FISH) break apart of *EWSR1* gene rearrangement was negative (Figure 3E). Next-generation sequencing (NGS) panel with 58 gene fusions (Cleveland Clinic Foundation panel) was performed, and CMTCT was detected. The final diagnosis was CMTCT. No recurrence or metastasis was found seven months after the initial resection.

## Discussion

The morphology and immunophenotype of our CMTCT case most closely resemble two other entities: cutaneous clear cell sarcoma of soft tissue (CCCSST) and dermal melanoma (DM). CCCSST can be either a primary dermal-based or metastatic tumor to the dermis. In contrast with CMTCT, CCCSST usually occurs in the lower extremities of young adults and exhibits aggressive behavior with high rates of recurrence and metastasis. Similar to CMTCT, CCCSST typically presents as a single small, well-circumscribed mass with occasional pigmentation, necrosis, and hemorrhage. Histologically, CCCSST is typically confined to the dermis with focal infiltration into the subcutis and rarely displays epidermotropism. It organizes in fascicles of uniform epithelioid or spindle cells with clear to pale eosinophilic cytoplasm and prominent nucleoli surrounded by delicate collagen fibers. Scattered wreath-like giant cells and mitotic figures are often seen, and occasionally necrosis can be present. CCCSST also expresses melanocytic markers. The hallmark molecular feature is *EWSR1* gene rearrangement with different partners, such as *ATF1 *(approximately 90% of cases) and, less commonly, *CREB1* and *CREM* [9]. In our case, the *EWSR1 *break apart FISH was negative, which helped rule out CCCSST.

DM can be primary or metastatic. Primary DM is extremely rare and usually occurs in elderly males with sun-damaged skin, typically presenting as a non-pigmented nodule. Metastatic DM is much more commonly seen, especially in patients with a history of melanoma. Melanoma is a great mimicker and can look similar to various tumors. Primary DM has the same molecular characteristics as metastatic DM. They have a high tumoral burden and usually have a specific driver mutation, most commonly *BRAF* (50% of melanomas). It is possible that some tumors with CMTCT were classified as melanoma due to a lack of molecular fusion testing. More neoplasms with specific fusions will be discovered as the available molecular studies continue to expand. Other differentials include clear cell tumors with melanocytic differentiation and MITF gene rearrangement [10], amelanotic cellular blue nevus, Spitz tumors, paraganglioma-like dermal melanocytic tumors (a heterogenous category that contains many of the above entities, and possibly PEComas (perivascular epithelioid cell tumor)), nerve sheath tumors, myoepithelial tumors, and soft tissue tumors with fibroblastic/fibrohistiocytic or myoid differentiation [11].

CMTCT shows reactivity with melanocytic markers such as S100 (focal to diffuse), SOX10 and MITF (diffuse), Melan-A, and HMB-45 (absent to focal). CMTCT can also be positive for p16, TRIM11, and NTRK1. To date, approximately six reported cases of CMTCT exhibit TrkA immunohistochemistry (IHC). FISH or microarray studies did not identify *NTRK* fusions or amplification in all six cases [1,11]. TrkA IHC was not performed on the remaining eight cases. The exact mechanism of this phenomenon is still unknown; it may reflect true* NTRK* gene over transcription by other mechanisms [1] or cross-reactivity with TrkA IHC. Similar findings of positive TrkA IHC have also been described in *BCOR* and *YWHAE *rearranged sarcomas [12]. TrkA IHC could be a potential time- and cost-efficient surrogate marker for CMTCT. However, the sensitivity and specificity of TrkA IHC in CMTCT are still unclear. Various immunohistochemical markers have been reported to be negative in CMTCT, including myoid, histiocytic, neuroendocrine, and neural markers, as well as pan-cytokeratin, EMA, Wilms' tumor 1 (WT1), anaplastic lymphoma kinase (ALK), ROS proto-oncogene 1 (ROS1), CD34, p63, calponin, CD99, and GLUT1.

*CRTC* (*CREB*-regulated transcription coactivator) belongs to a family of a gene comprising three members (CRTC1, CRTC2, and CRTC3). The *CRTC1* gene, also known as *TORC1, MECT1*, and *WAMTP1*, is located on the short arm of chromosome 19. CRTC1 translocates to the nucleus and mediates transcription of *CREB *(cAMP response element-binding) target genes integral to cell-cycle control, cellular proliferation, and differentiation [6,13]. *CRTC1 *expression is limited to a few normal tissues, such as the brain, skeletal muscles, liver, and salivary glands, playing key roles in memory, metabolism, and morphogenesis [14]. *CRTC1 *and its isoforms have been implicated in oncogeneses, such as colon adenocarcinoma [15] and a non-*CREB*-mediated pathway [13]. Similarly, *CRTC1::MAML2 *fusions have been described in mucoepidermoid carcinoma of various sites [16], whereas *CRTC1::SS18* fusion has been implicated in a subset of undifferentiated small round blue cell sarcomas [17] CMTCT have only been reported in CMTCT [6].

Tripartite motif-containing 11 (*TRIM11*) is located on the long arm of chromosome 1 and belongs to a gene family encoding E3 ubiquitin ligase protein, whose main role is directing misfolded proteins toward proteasomes for degradation [1]. *TRIM11* overexpression is found in various malignancies. Studies have shown that *TRIM11* knockout in ovarian cancer cells leads to increased apoptosis or cessation in cell cycle progression [18]. On the contrary, *TRIM11* overexpression in lymphoma promotes proliferation via the beta-catenin/Wnt pathway [19]. *TRIM11* upregulation has also been correlated with increased angiogenesis, cellular invasiveness, and proliferation, correlating to poor clinical outcomes and advanced disease stages [18,20].

**Figure 4:**
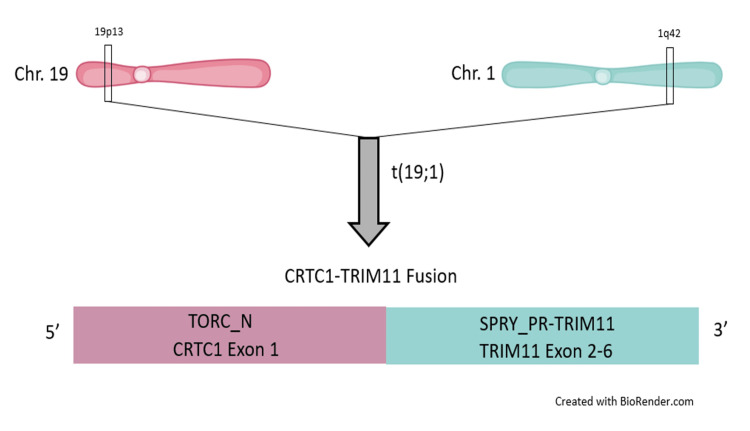
Schematic diagram of CRTC1-TRIM11 fusion.

The *CRTC1::TRIM11* chimeric protein (t(19;1)(p13.11;q42.13)) includes the TORC_N domain of the *CRTC1* protein merged with the SPRY_PRY-TRIM domain of *TRIM11* (Figure 4). The CMTCT has been detected in various studies by RNA sequencing, reverse transcription-polymerase chain reaction (RT-PCR)/direct sequencing, and FISH. As a result of fusion, the potential specific protein-protein interaction site of *TRIM11* deemed crucial for protein degradation is deleted. Therefore, it is possible that the fusion of *CRTC1* and *TRIM11 *could result in a loss of function of *TRIM11* in its role of protein degradation, leading to tumorigenesis [7]. However, the exact mechanism of the* CRTC1::TRIM11 *transcript remains to be elucidated.

## Conclusions

To our knowledge, there are not many CMTCT cases reported in the literature, this emerging entity remains largely unknown, and our case is the first case arising in a digit. This case contributes to the growing knowledge surrounding this newly described entity. CMTCT is a novel entity that expands the differential for the diagnostically challenging cutaneous tumors with melanocytic differentiation. In difficult cases, molecular studies are required to differentiate this entity from tumors with more aggressive behavior, such as CCCSST and melanoma.
